# Colon perforation due to collagenous colitis: A case report

**DOI:** 10.1002/ccr3.7862

**Published:** 2023-09-13

**Authors:** Kei Ito, Keita Nakatutumi, Yuko Oofuti, Yasuhiro Otomo

**Affiliations:** ^1^ Trauma and Acute Critical Care Center Tokyo Medical and Dental University Hospital of Medicine Tokyo Japan; ^2^ Department of Acute Critical Care and Disaster Medicine, Graduate School of Medical and Dental Sciences Tokyo Medical and Dental University Tokyo Japan

**Keywords:** collagenous colitis, colonic perforation, lansoprazole, microscopic colitis, PPI

## Abstract

Collagenous colitis (CC) is generally benign, and serious complications are rare. It is important to note that spontaneous perforation of CC is a possible complication. In the case of colon perforation of unknown origin, CC should be considered.

## Graphical abstract

A rare complication of collagenous colitis.

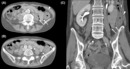

## 1 BACKGROUND

Collagenous colitis (CC) is a microscopic colitis characterized by watery diarrhea without bloody stool.^1^ CC often occurs in middle‐aged females. Smoking and the use of medication such as proton pump inhibitors (PPI) and nonsteroidal anti‐inflammatory drugs (NSAIDs) are known risk factors.^2^ The main treatment for CC is the discontinuation of medication and smoking cessation. Medication therapies such as glucocorticoids, budesonide, and prednisone are added to the active state of the disease.^3^ Very few patients are refractory to drug treatment and require surgical treatment.^4^

Herein, we report the case of a 58‐year‐old female who underwent emergency surgery for colonic perforation due to CC.

### 1.1 Case presentation

**FIGURE 1:**
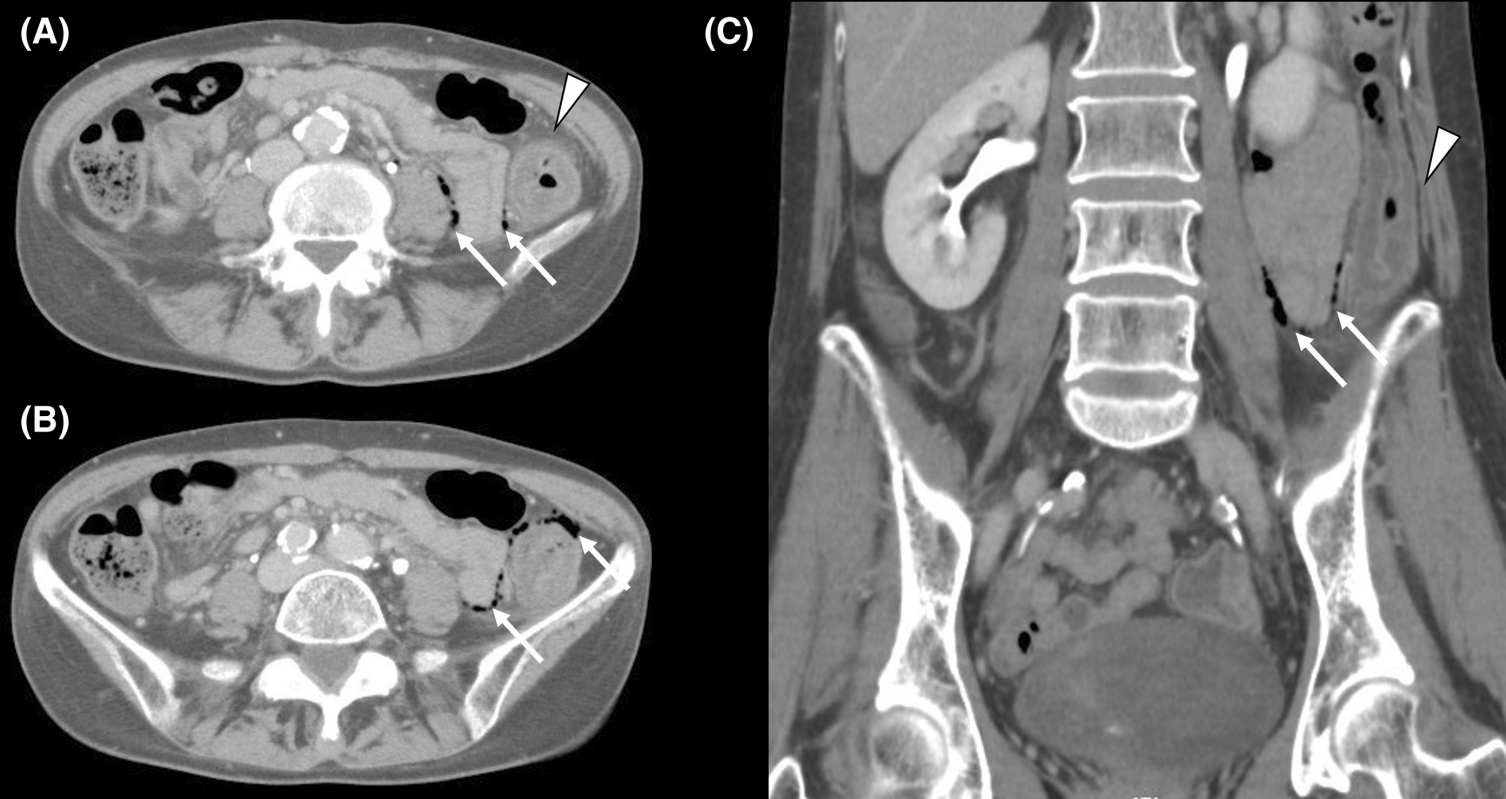
The initial CT scan reveals thickening of the wall of the descending colon (A, C arrowhead) and free air around the colon and in the retroperitoneum (B, Carrow). CT, computed tomography.

**FIGURE 2:**
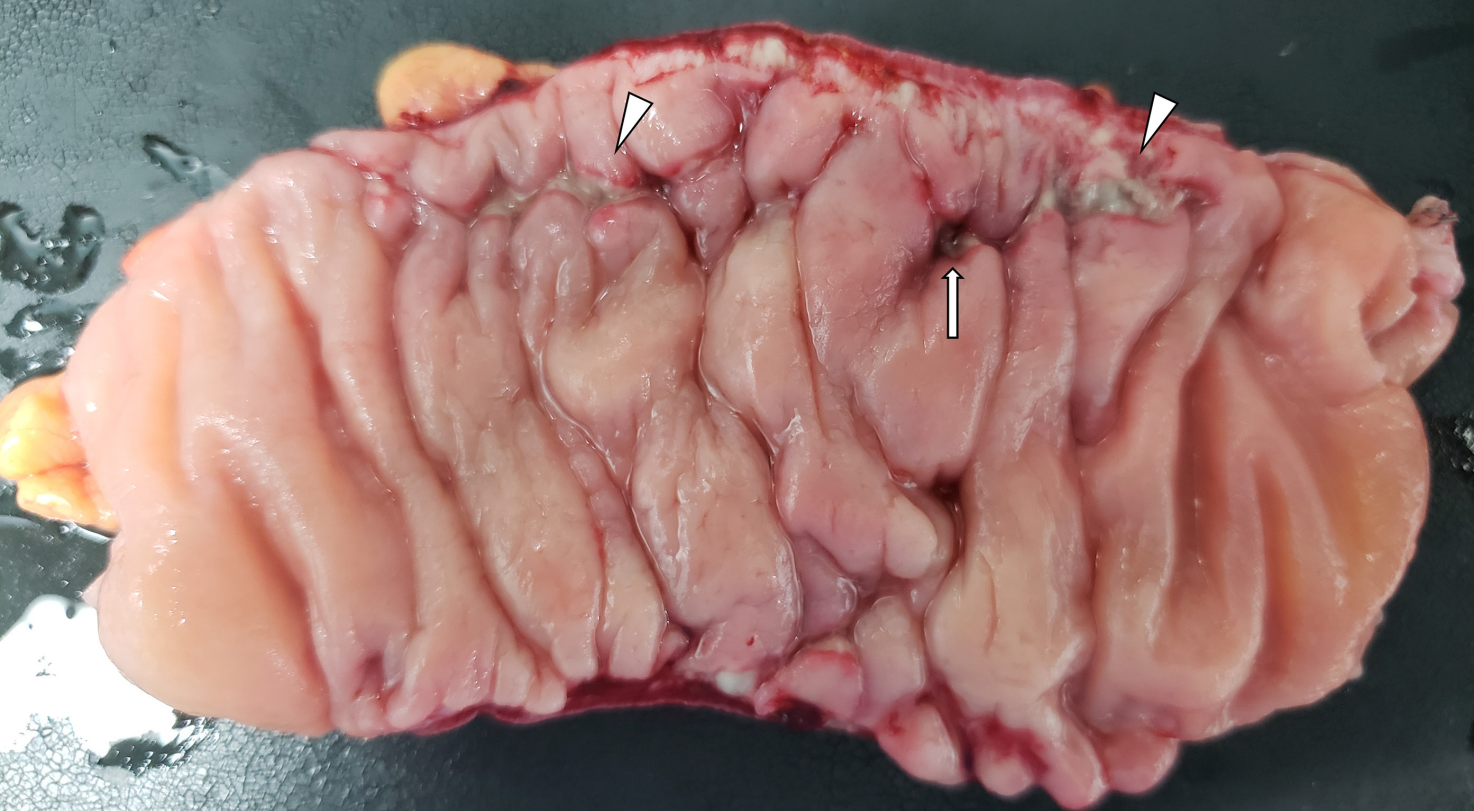
The macroscopy of the resected colon reveals multiple linear ulcers called “mucosal tears” in collagenous colitis (arrowhead), and an arrow indicates the perforation. This revealed no diverticulum or tumor in the colon.

**FIGURE 3:**
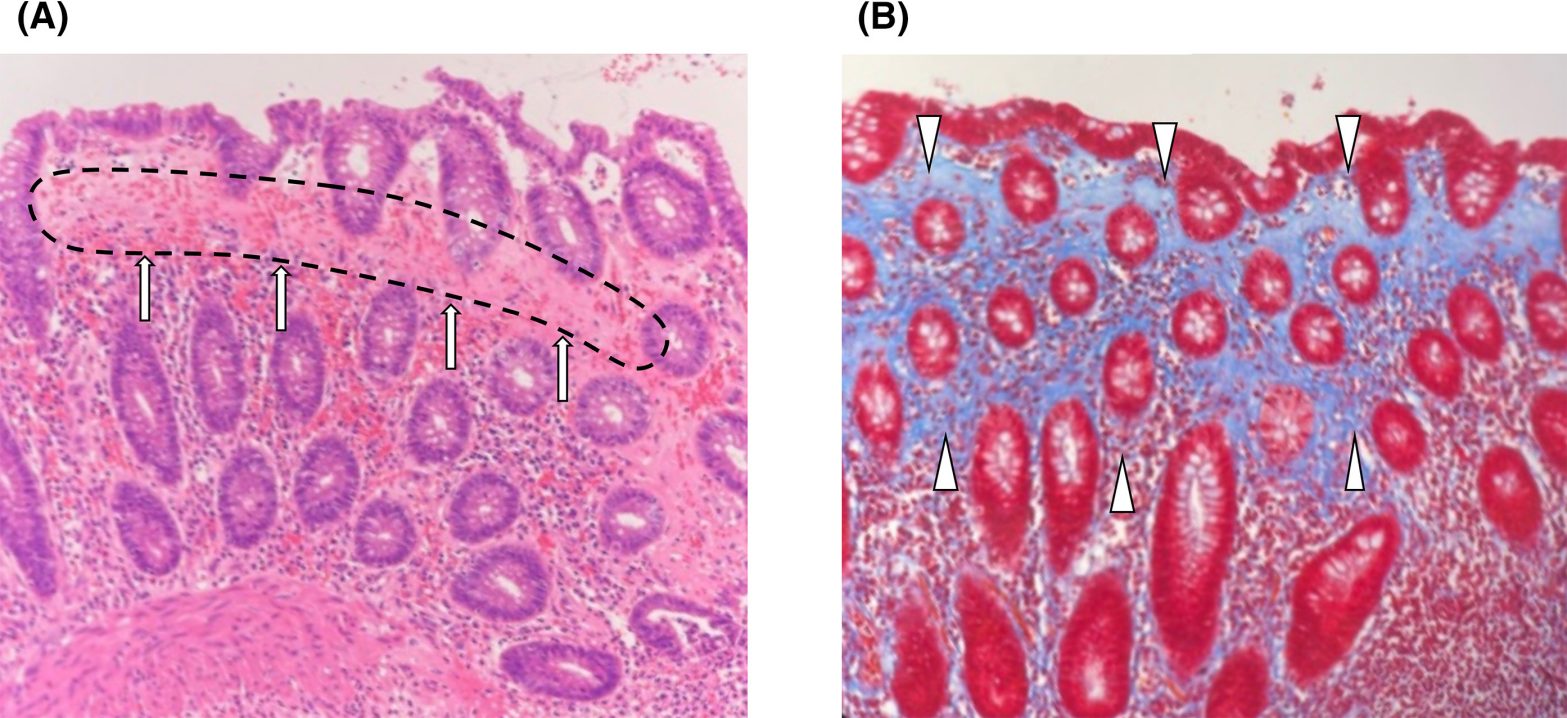
Histopathological examination revealed a thick subepithelial collagen band by HE stains (A, arrows and dotted line). This collagen band is stained blue by Masson trichrome stain (B arrowheads). HE stains, Hematoxylin and eosin stain.

A 58‐year‐old female, complaining of constant abdominal pain presented to the hospital. There were no symptoms of diarrhea. Her past medical history consisted of hypertension, insomnia, constipation, and a 38‐year smoking history. Her medications at presentation were lansoprazole, irbesartan, amlodipine besilate, and etizolam. She had been on this medication for several years. As computed tomography (CT) detected colon perforation, she was transferred to our hospital for surgery and intensive care. Her vital signs on arrival were as follows: blood pressure, 114/60 mmHg; pulse rate, 60/min, oxygen saturation, 99% on room air, and body temperature 37.2°C. Physical examination revealed left lower abdominal tenderness and rebound tenderness. The laboratory test results were as follows: white blood cell counts of 11,500/μL; C‐reactive protein, 1.95 mg/dL. An abdominal CT scan showed a thickened descending colon, accompanied by free air (Figure 1). An emergency laparotomy was performed. A few purulent ascites were found in the abdomen. Although no obvious perforation site was identified, the descending colon was thickened and suspected to be the cause of perforation. Thus, partial colon resection and covering ileostomy were performed. The anastomosis was performed by functional end‐to‐end anastomosis. Three 19Fr drains were placed in the abdomen, and the operation was completed. Macroscopic examination of the resected colon revealed a longitudinal ulcer and no diverticulum (Figure 2). The length of the ulcer was 7.5 cm. Histopathological examination revealed a colonic subepithelial collagen band in the superficial epithelium on hematoxylin and eosin staining, which showed an edematous appearance of the submucosa and a generalized neutrophilic infiltrate, and CC was diagnosed (Figure 3). Antibiotic therapy was continued for 5 days postoperatively. Drains were removed on postoperative day 5. The patient was discharged without complications on postoperative day 9. There was no diverticulum or inflammatory bowel disease on histopathological examination. PPI therapy was thought to be the cause of CC, and lansoprazole was changed to famotidine. After being discharged from the hospital, she had no recurrence of abdominal pain or new‐onset watery diarrhea. Ileostomy closure was performed three months after discharge.

## 2 DISCUSSION

CC is a type of the microscopic colitis, characterized by chronic, nonbloody, watery diarrhea,^1^ and it occurs in middle‐aged women. In the European guidelines on microscopic colitis, the chronic or frequent use of PPI, NSAIDs, or serotonin reuptake inhibitors is associated with an increased risk of CC.^2^ In particular, PPI use is strongly associated with CC especially when used continuously for 4–12 months. PPI use is the most reported drugs related to CC. More cases of CC due to lansoprazole have been reported than any other PPI.^5^, ^6^, ^7^, ^8^, ^9^, ^10^, ^11^, ^12^ Therefore, in the present case, the cause of CC was determined to be PPI.

Colonic perforation is a rare complication of CC. The leading cause is iatrogenicity, such as colonoscopy, and most of these perforations occur in the right‐side colon.^13^ Linear ulcerations and deep mucosal tears observed mainly on the right side of the colon, are considered to be a risk of the perforation.^14^, ^15^ Furthermore, spontaneous perforation in the present case is rare. Mori et al. reported seven cases of spontaneous colonic perforation associated with CC.^16^ All the patients underwent colon resection and recovered well. Interestingly, these cases and the present case occurred in the descending colon, while spontaneous colon perforation unrelated to CC commonly occurs in the sigmoid colon.^17^ Therefore, it might be useful to consider the possibility of CC if a descending colon perforation of unknown origin is observed.

Watery diarrhea is the main symptom of CC, but almost 10% of patients with CC have no typical symptoms.^18^ The present case also had no history of diarrhea. The mechanism of diarrhea is associated with surface injury of the mucosal membrane due to inflammatory mediators in the luminal propria.^19^ Although the present case did not state complaints of diarrhea, histopathological examination showed an edematous appearance of the submucosa and a generalized neutrophilic infiltrate. This can lead to spontaneous colonic perforation of the colon.

The criteria for the histological diagnosis of CC are thickened subepithelial collagenous band ≧10 μm.^20^ In the present case, a thickened subepithelial band was also found around the perforation site. There were no clinical findings clinical findings indicating diverticulitis, inflammatory bowel disease, or tumors. Furthermore, the present case took PPIs regularly. Therefore, we concluded that CC caused colon perforation in the present case.

## 3 CONCLUSION

We encountered a rare complication of CC. Only a few cases were described in medical literature so far. In the case of colon perforation in an uncommon site, without diverticulum or tumor lesion, and when no obvious cause of perforation can be identified from the specimen, it is important to examine the patient's background, particularly medication history, and symptoms in detail to identify the etiology and consider CC as a potential cause. In addition, when performing an endoscopic examination on patients suspected of having CC, there is a risk of perforation, so caution is required.

## AUTHOR CONTRIBUTIONS

**Kei Ito:** Writing – original draft. **Keita Nakatutumi:** Writing – review and editing. **Yuko Oofuti:** Visualization; writing – original draft. **Yasuhiro Otomo:** Supervision; writing – review and editing.

## FUNDING INFORMATION

None.

## CONFLICT OF INTEREST STATEMENT

The authors declare no conflicts of interest.

## CONSENT

Written informed consent was obtained from the patient.

## Data Availability

Data supporting this study are available from the corresponding author upon reasonable request.
